# Ethanol locks for the prevention of catheter-related bloodstream infection: a meta-analysis of randomized control trials

**DOI:** 10.1186/s12871-018-0548-y

**Published:** 2018-07-24

**Authors:** Peng Zhang, Jun-Hao Lei, Xin-Jun Su, Xing-Huan Wang

**Affiliations:** Department of Urology, Zhongnan Hospital, Wuhan University, 169 Donghu load, Wuchang district, Wuhan, 430071 Hubei People’s Republic of China

**Keywords:** Catheter-related bloodstream infection, Ethanol lock, Meta-analysis

## Abstract

**Background:**

Current evidence regarding the efficacy of ethanol locks in preventing catheter-related bloodstream infection (CRBI) is inconclusive.

**Methods:**

Electronic databases, including PubMed, Web of Science, Embase, and the Cochrane Library (until April 2018),were systematically searched for relevant studies. Two reviewers independently screened the retrieved records and identified RCTs that met the inclusion criteria. Relevant data were extracted for pooled analyses using Review Manager 5.3 software. Subgroup analysis was performed according to the study quality, duration of the ethanol lock, disease type and CRBI definition. Eggs’ method was applied to detect publication bias. Sensitivity analysis was conducted to check the stability of the meta-analysis results.

**Results:**

Ten RCTs involving 2760 patients were included in the analysis. The overall pooled result indicated that ethanol locks significantly reduced the incidence of CRBI (RR 0.66, 95% CI 0.51–0.86). Subgroup analysis suggested that an ethanol lock significantly decreased the incidence of CRBI in patients with hematological diseases (RR 0.50, 95% CI 0.31–0.80). An ethanol lock significantly reduced the incidence of CRBI in a2-hour ethanol lock group (RR 0.49, 95% CI 0.33–0.73). The meta-analysis showed that an ethanol lock significantly reduced the incidence of CRBI according to analysis of high-(RR 0.66, 95% CI 0.47–0.94) or low-(RR 0.66, 95% CI 0.46–0.95) quality studies. Meta-analysis of studies with a strict CRBI definition showed that an ethanol lock can significantly prevent CRBI (RR 0.61, 95% CI 0.42–0.89). The results of sensitivity analysis suggested that the pooled result was stable. Meta-analysis of adverse events showed that an ethanol lock did not increase the incidence of thrombosis (RR 1.05, 95% CI 0.51–2.18) or mortality (RR 0.99, 95% CI 0.90–1.08) but did result in increased nausea (RR 1.54, 95% CI 1.01–2.35), dizziness (RR 4.21, 95% CI 2.40–7.39),elevated blushing rates (RR 3.27, 95% CI 2.05–5.22) and altered taste rates (RR 2.61, 95% CI 1.93–3.54).

**Conclusions:**

An ethanol lock may play a role in the prevention of CRBI, especially in immunocompromised patients with hematological diseases.

**Electronic supplementary material:**

The online version of this article (10.1186/s12871-018-0548-y) contains supplementary material, which is available to authorized users.

## Background

Tunneled central venous catheters(CVCs) are widely used for long-term venous access to deliver blood and its products, chemotherapy and parenteral nutrition [1]. However, despite improved international guidelines on CVC placement and catheter care, the use of CVCs carries a high risk of developing catheter-related bloodstream infection (CRBI) [2]. Furthermore, CRBIs are related to increased healthcare costs, morbidity, hospitalization and death [3].

There are many ways to reduce CRBIs, including antimicrobial lock solutions, catheter care procedures, and agents that reduce nasal colonization of *Staphylococcus aureus*, and one meta-analysis showed that antimicrobial lock solutions significantly reduce the risk of CRBI [4]. Overall, ethanol locks are considered a promising lock solutions because they are inexpensive, universally available, and effective against a broad spectrum of bacteria and fungi [5]. Nevertheless, study results to date on ethanol locks are controversial.

For example, Bertrand Souweine et al. observed that a 2-min ethanol lock does not decrease the frequency of infection of dialysis catheters(DCs) in intensive care unit (ICU) patients [6]. A randomized pilot study showed that a 30% ethanol/4% sodium citrate appears to prevent CRBI and may improve catheter survival compared to heparin [7], and a randomized controlled multi-center trial showed that ethanol locks can prevent CRBI in pediatric oncology patients [8]. However, ethanol lock therapy has not been observed to affect patients after major heart surgery (MHS) [9].

Here, we present the results of a meta-analysis to investigate the association between ethanol locks and CRBI.

## Methods

This study was performed according to the preferred reporting items of the systematic review and meta-analysis (PRISMA) guidelines.

### Systematic search strategy

We conducted an electronic search of the PubMed (1966 to April2018), Embase (1974 to April2018), Science Citation Index (1974 to April 2018) and Cochrane (April 2018) databases for relevant studies on the efficacy of ethanol locks in preventing CRBI. The two keywords used to search the above electronic databases were ‘ethanol lock’ and ‘infection.’ All reference sections of eligible studies were hand-reviewed for potential inclusion, and no limits on language were imposed.

### Eligibility criteria

We included studies if they met the following criteria: (1) study participants were patients with indwelling central venous catheters,(2) the intervention group received ethanol locks and the control group heparin/NaCl locks, and (3) the studies were randomized controlled trials (RCTs).

### Study selection and data extraction

Two reviewers independently screened and assessed titles and abstracts to confirm whether the inclusion criteria were met. Data, including study characteristics (title, publication time, and sample size), detailed information in the PICOS approach (participant, intervention, comparison, outcomes, and study design), and other characteristics, were extracted by two authors using standard data extraction forms. Where necessary, the authors of the original studies were contacted for missing information.

### Methodological quality assessment

The Cochrane Collaboration tool for assessing the risk of bias was used to evaluate the methodological quality of each included RCT. There were seven items for assessing bias including random sequence generation (selection bias), allocation concealment (selection bias), blinding of participants and personnel (performance bias), blinding of outcome assessment (detection bias), incomplete outcome data (attrition bias), selective reporting (reporting bias) and other biases. Each item was categorized as a low risk of bias, an “unclear” (either lack of information or uncertainty about the potential for bias) risk of bias, or a high risk of bias under the guidelines in the Cochrane Handbook.

### Data synthesis and analysis

The meta-analysis was performed using Review Manager 5.3 software based on PRISMA guidelines. Heterogeneity was assessed by examining the clinical characteristics of the included studies and by formal statistical χ^2^and I^2^tests. For main outcomes (incidence of CRBI), Mantel-Haenszel estimates with a random-effects analytical model (due to the considered between-trial heterogeneity) were used to calculate relative risks (RRs) and their 95% confidence intervals (CIs). The funnel plot methods of Egger’s test were used to assess publication bias. We performed subgroup analysis according to study quality, duration of the ethanol lock, disease type and CRBI definition. Sensitivity analysis was conducted to determine the stability of the meta-analysis results using Stata 12.0 software.

## Results

### Characteristics of the included studies

**Fig. 1:**
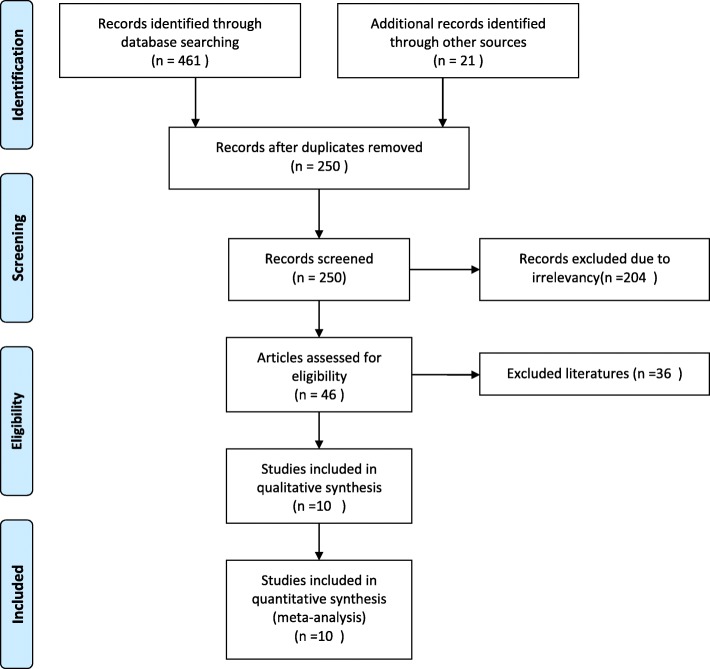
PRISMA flow diagram

The initial results of databasesearchingproduced461 records and 10 studies [6–8, 10–16] that met the inclusion criteria and were ultimately included after screening and reviewing by the authors. The selection flowchart is shown in Fig. 1. Reasons for the exclusion of 36 studies in the literature screening process are presented in Additional file 1.

The characteristics of the 10 included trials are listed in Table 1. A total of 2760 patients were included in the meta-analysis, among whom1396receivedanintervention with ethanol locks. Three studies [10, 11, 16] included only patients with hematological diseases, and 4 included hemodialysis patient [6, 7, 13, 15]. Pediatric oncology patients were included only in one study [8], and the remaining two studies involved home parenteral nutrition patients [12] or those after major heart surgery [9].

**Table 1:** Characteristics of included studies

NO.	Country	Sample size(T/C)	Gender(T/C, %)	Age(T/C)	Inclusion criteria	Exclusion criteria	Intervention	Control	Diagnosis of CRBSI	Catheter types	Follow up
Bradley R.S. 2017 [12]	USA	18/20	M(33/30)	49/52	Adult patients providing consent, non-medicare insurance, or medicare insurance with a supplementary insurance, anticipated duration on home parenteral nutrition(HPN) > 3 m not previously on HPN at Mayo Clinic or elsewhere, patients with single lumen, silicone Hickman ® catheters, and no known alcohol addiction	Failure to provide consent, medicare insurance without supplemental private insurance, patients with a catheter type other than a single lumen Hickman ®, patients who were anticipated to be on HPN for less than three months, pregnant patients, patients who have previously proven addiction and/or dependence to alcohol	3 ml 70% ethanol lock until next PN infusion	Heparin lock plus saline infusion	(1)Bacteremia or fungemia in a patient who had an intravascular device and > 1 positive blood culture result obtained from the peripheral vein; (2)Clinical manifestations of infection (e.g., fever, chills, and/or hypotension), and no apparent source for blood stream infection other than the central venous catheter.	Single lumen, silicone Hickman ® catheters	NA
Lavern M.V. 2016 [7]	Canada	20/19	M(60/47)	63/62.3	Eligible participants were 18 years of age or older with end-stage renal disease and planned vascular access with a catheter or current hemodialysis patients requiring exchange of an existing catheter	(1) they were critically ill in the ICU setting, (2) had acute kidney injury and were unlikely to require prolonged vascular access, (3) had a maturing or planned arteriovenous fistula/graft creation within 2 months, or (4) planned antibiotic treatment courses lasting longer than 4 weeks from the date of the new catheter insertion.	2.5 ml 30% ethanol/4% sodium citrate for a 6-month period	Heparin 1000 units/mL	Two or more positive blood cultures of the same organism (species, antibiogram) from any source (peripheral or intravascular device cultures) from a patient with clinical and microbiologic data suggesting no other source for the bacteremia except the intravascular device.	Inserted dual-lumen tunneled, cuffed catheters made of carbothane, an alcohol-resistant polymer with silicone extensions (Tal Palindrome, Tyco Healthcare Kendall/Covidien, Mansfield, MA, USA)	If an outcome occurred, an additional 30 days for safety was added
Bertrand 2015 [6]	France	730/730	M(60.8/61.1)	65/66	Patients required insertion of DCs with an expected duration of use longer than 48 h in ICU	Ethanol intolerance and pregnancy	60% wt/wt EL locks for 2 min	0.9% saline lock	In patients with one or more blood cultures positive for coagulase-negative staphylococci, identity of pulse-field gel electrophoresis patterns in the catheter tip and blood cultures was required for a diagnosis of CRBSI	A nontunneled, nonantimicrobial-impregnated double-lumen dialysis catheters (DC)	Until death or 48 h after ICU discharge
Reineke A 2015 [8]	Netherlands	153/154	M(58/56)	9.8/7.8	Paediatric oncology patients (1–18 years) with a newly inserted, tunnelled central venous catheters (CVC)	≤1 year at diagnosis, a primary immunological disorder, an ethanol allergy or a CVC inserted in a vessel with previously confirmed thrombosis	70% ethanol locks for 2 h	100 IU/ml heparin locks	At least one of the following criteria: (1) recognised pathogen cultured from ≥1 blood cultures, not related to an infection at another site (2) Clinical manifestations of infection and a common skin microorganism (such as coagulase-negative staphylococci (CoNS), diphtheroids, Bacillus spp., or micrococci) cultured from ≥2 blood cultures drawn on separate occasions	Tunnelled CVC(port-a-cath (PAC) or Broviac)	Time to CABSI or death due to CABSI, during a maximum follow-up period of six months
L.J. Worth 2014 [16]	Australia	42/43	M(28/24)	47.0/48.1	Patients with haematological malignancy or planned BMT were eligible for enrolment at time of insertion of a dual lumen, non-antibiotic-impregnated, tunnelled, cuffed, intravascular catheter (Hickman catheter) into subclavian or internal jugular veins, where the intended period of catheterization was 30 days.	NA	70% ethanol locks for 2 h	Heparinized saline	A positive blood culture with a recognized pathogen or common commensal, with confirmation of infection by isolation of the same organism following culture of catheter tip, or a differential time to positivity for centrally and peripherally drawn blood cultures of 2 h	A dual lumen, non-antibiotic-impregnated, tunnelled, cuffed, intravascular catheter (Hickman catheter)	Until a device-related bloodstream infection occurred, or planned study end-date
Sishir 2014 [15]	India	35/35	NA	NA	Hemodialysis population	NA	70% ethanol lock for 20 min	Heparin lock (1000 U/ml)	NA	Double lumen polyurethane hemodialysis cathete	NA
Mara 2014 [14]	Spain	113/87	M(55/54)	67.3/65.2	Recent MHS admission with Central Vascular Catheters (CVC) inserted >48 h; Age >18 years; No evidence or suspicion of CRBSI at enrolment: No signs of infection neither general nor at catheter site entrance	Allergy or intolerance to ethanol or chronic liver disease;Pregnancy	70% ethanollock for 2 h	Conventional catheter-care	Microbiologically proven CRBSI considered when the same microorganism was recovered from blood and a catheter tip within less than 8 days.	Conventionalcatheter	Enrolled patients were prospectively followed for the occurrence of CR-BSI until catheter withdrawal, hospital discharge or death
Jennifer K 2012 [13]	Australia	25/24	M(52/46)	52/64	Adults > 18 years, the presence of a tunnelled intravenous catheter and the ability to give informed consent	Pregnancy or breast feeding, religious or personal objection to the use of ethanol, intolerance of ethanol, and a history of an exit site, tunnel or blood stream infection associated with the current catheter.	70% ethanol for 48 h	Thrice weekly standard heparin locks(Heparin sodium 5000 U/Ml)	(1)Positive blood cultures for the presence of bacteria with or without Clinical manifestations of infection	A tunnelled central venous catheter	NA
Lennert Solbbe 2010 [11]	Netherlands	226/222	M(57.5/56.3)	51.7/49.8	Eligible study-participants were all consecutive adult (age>17 years) hematology patients with a tunnelled silicone CVC, inserted in the preceding 72 h before study-entry	Patients with an alcohol-intolerance or concomitant treatment with metronidazole	70% ethanol lock for 15 min per day	0.9% NaCl	A positive central or peripheral blood culture; For (coagulase-negative staphylococci) or other skin-colonizers, 2 blood cultures had to be positive when no peripheral cultures were available [19]	A tunnelled silicone CVC	NA
Sanders 2008 [10]	New Zealand	32/28	M(53/57)	52.4/47.2	An age >18 years or older and admission as an inpatient to receive intensive chemotherapy likely to produce neutropenia (<0.5 × 109 L) for the treatment of haematological disease, including haematopoietic stem cell transplantation.	Abnormal liver function tests or a history of alcohol abuse	70% ethanol for 2 h	Control	The culture of a recognized pathogen from one or more blood cultures, unrelated to infection at another site [18].	Identical dual lumen Hickman central venous catheters	The study period ended with either diagnosis of CABSI, removal or failure of catheter, discharge from hospital, death or end of study period after an arbitrary 30 days.

### Risk of bias

**Fig. 2:**
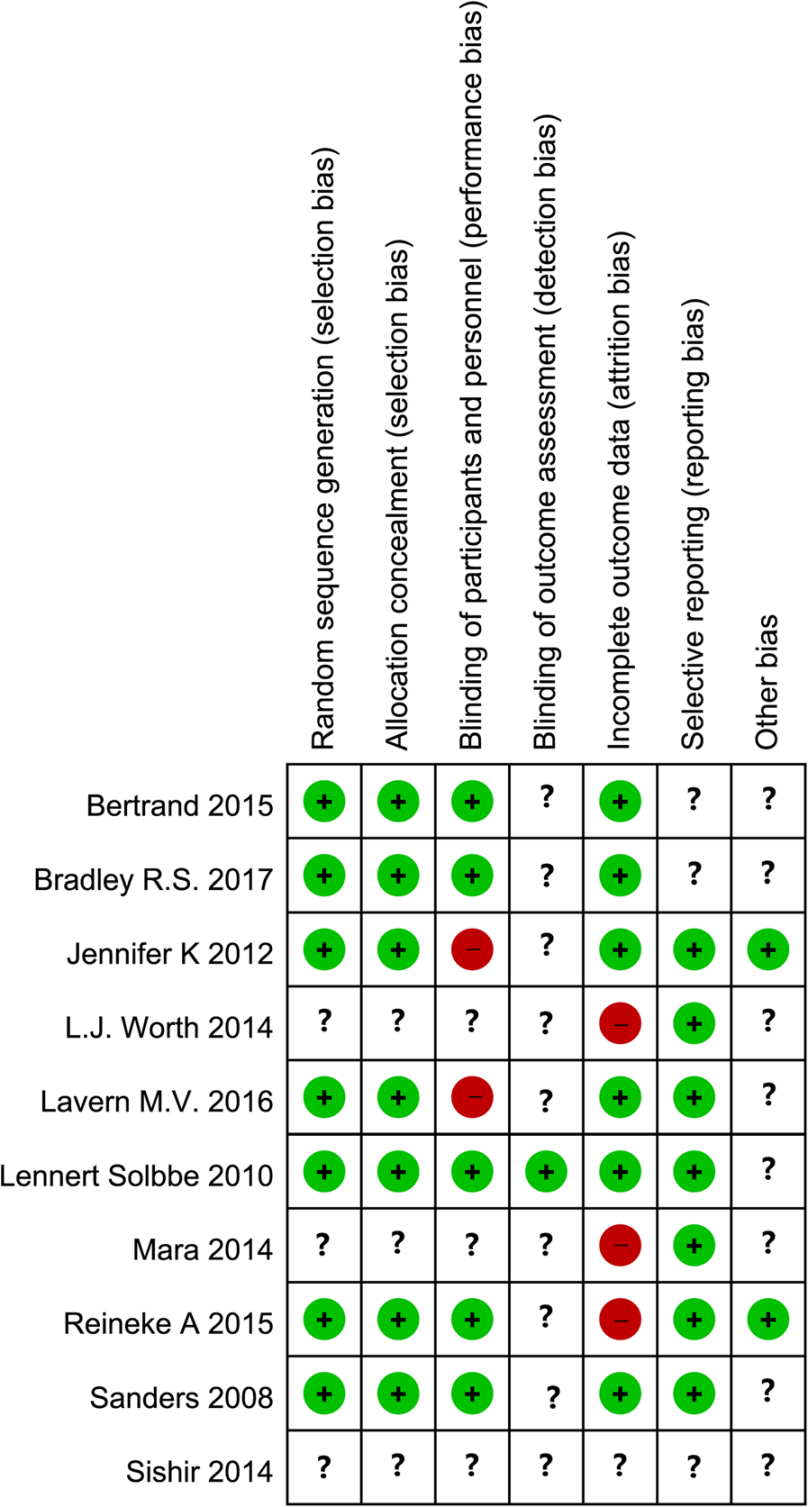
Risk of bias assessment for the included RCTs

There were seven studies [6–8, 10–13] that were considered to have a low risk of bias for “Random sequence generation” and “Allocation concealment.” “Blinding of participants and personnel” was judged to have a low risk of bias in five studies [6, 8, 10–12] and a high risk in two studies [7, 13]. There was only one study that was deemed to have a low risk of bias for the item “Blinding of outcome assessment” [11]. For “Incomplete outcome data”, six studies had a low risk of bias [6, 7, 10–13] and three a high risk [8, 14, 16]. There were seven studies [7, 8, 10, 11, 13, 14, 16] that could be judged as having a low risk of bias in the item “selective reporting.” The risk of bias assessment results are shown in Fig. 2.

### CRBI

**Fig. 3:**
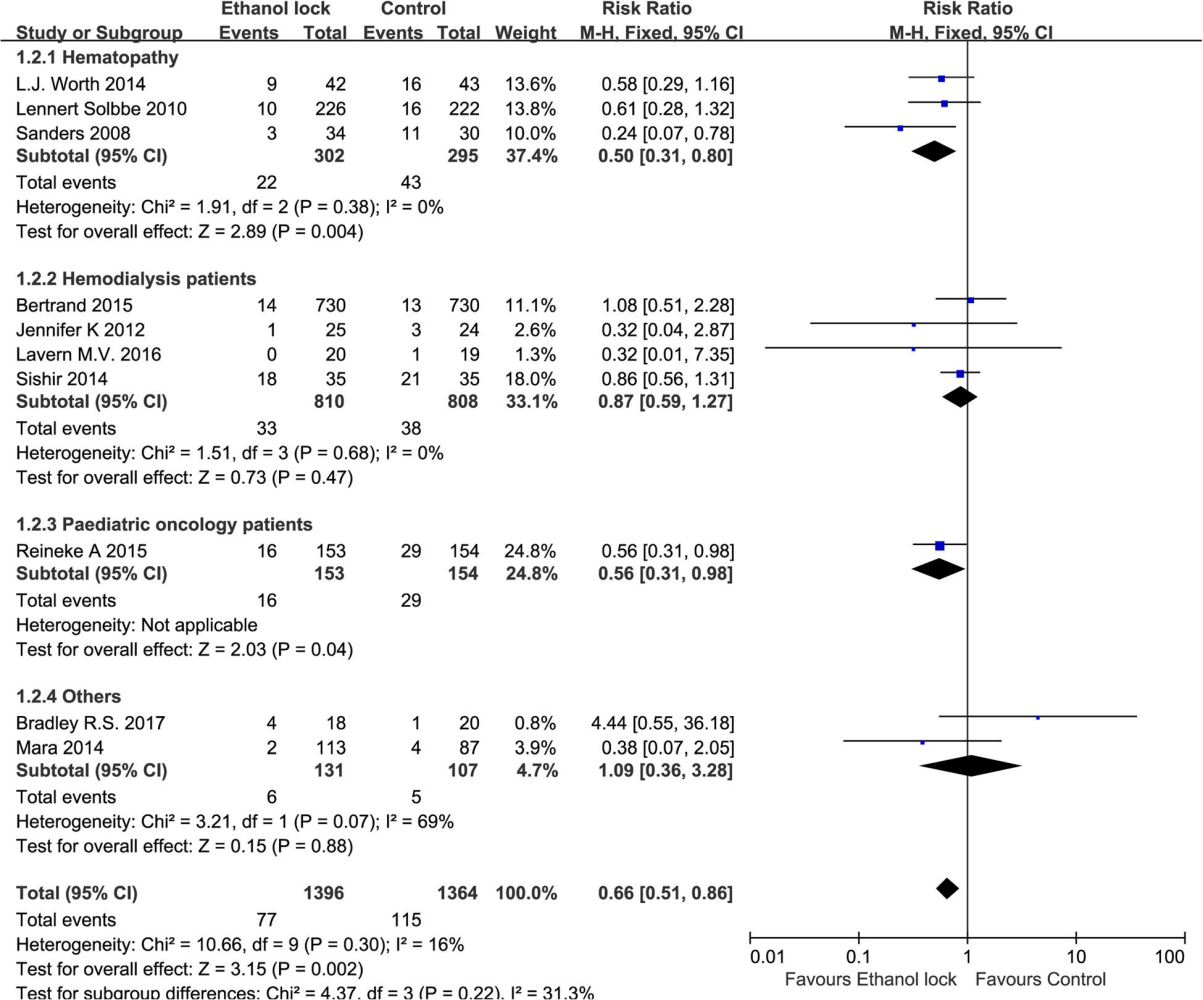
Forest plot for subgroup analysis of the incidence of CRBI according to patients with different diseases (RR, relative risk; CI, confidence interval)

Definitions of CRBI among the included studies are shown in the Table 1, and a positive blood culture was necessary to diagnose CRBI [17, 18]. All included studies reported the incidence of CRBI. The total pooled results showed that there was a significant difference between ethanol locks and conventional catheter-care (RR 0.66, 95% CI 0.51 to 0.86), without significant heterogeneity (I^2^ = 16%, Fig. 3). The pathogens involved in the reported infections are shown in Table 2.

**Table 2:** the pathogens involved in the infections

NO.	Ethanol lock(n)	Control(n)
Bradley R.S. 2017 [12]	Candida species(2); Staphylococcus species(1); Escherichia coli plus Klebsiella species plus Pseudomonas(1).	Unidentified gram positive cocci
Lavern M.V. 2016 [7]	NA	Klebsiella Pneumonia
Bertrand 2015 [6]	Staphylococcus epidermidis(20) Staphylococcus aureus(2) Enterococcus species (0) Other coagulase negative Staphylococci(31) Other Gram-positive (5) Escherichia coli (2) Proteus species (0) Pseudomonas aeruginosa (10) Enterobacterspecies (2) Other Gram-negative (2) Fungi (5) Polymicrobial(20)	Staphylococcus epidermidis(9) Staphylococcus aureus(0) Enterococcus species (1) Other coagulase negative Staphylococci(27) Other Gram-positive (6) Escherichia coli (1) Proteus species (2) Pseudomonas aeruginosa (9) Enterobacterspecies (0) Other Gram-negative (3) Fungi (3) Polymicrobial(17)
Reineke A 2015 [8]	Staphylococcus epidermidis(2) Other coagulase-negative Staphylococc(4) Staphylococcus aureus(0) Streptococcus parasanguis(1) Other alpha-haemolytic streptococci (1) Enterococcus faecalis(0) Bacillus sp.(0) Streptomyces sp.(0) Escherichia coli(1) Citrobacter freundii(1) Brevundimonas vesicularis(1) Gram-negative rod (1) Polymicrobial(3) Candida sp.(1)	Staphylococcus epidermidis(1) Other coagulase-negative Staphylococc(8) Staphylococcus aureus(2) Streptococcus parasanguis(0) Other alpha-haemolytic streptococci (0) Enterococcus faecalis(1) Bacillus sp.(2) Streptomyces sp.(1) Escherichia coli(3) Citrobacter freundii(0) Brevundimonas vesicularis(0) Gram-negative rod (0) Polymicrobial(0) Candida sp.(2)
L.J. Worth 2014 [16]	Coagulase-negative Staphylococcus spp. (3), Staphylococcus aureus (1), Listeria monocytogenes (1), Klebsiella pneumoniae (2), Escherichia coli (1), Pseudomonas aeruginosa (1), E. coli (2)and C. glabrata (1).	Coagulase-negative Staphylococcus spp. (7), Staphylococcus aureus (1), Enterococcus faecium (1), Klebsiella pneumoniae (2), Escherichia coli (1), Pseudomonas aeruginosa (1), Enterobacter cloacae(1), E. coli and E. faecium (1), and Candida parapsilosis (1).
Sishir 2014 [15]	NA	NA
Mara 2014 [14]	Gram positive cocci(0) Enterobacteriaceae(2) Gram negative non-fermenting rods(0) Fungi(0)	Gram positive cocci(1) Enterobacteriaceae(2) Gram negative non-fermenting rods(1) Fungi(0)
Jennifer K 2012 [13]	Staphylococcus aureus(1)	Staphylococcus aureus(1) Enterobacter cloacae(1) Staphylococcus hominis (1)
Lennert Solbbe 2010 [11]	n = episodes Coagulase-negative staphylococci.(49) Other skin colonizers(2) Staphylococcus aureus(2) Other gram-positive cocci(12) Gram-negatives(4) Polymicrobial(20) Yeasts(2)	n = episodes Coagulase-negative staphylococci.(57) Other skin colonizers(2) Staphylococcus aureus(3) Other gram-positive cocci(10) Gram-negatives(5) Polymicrobial(13) Yeasts(1)
Sanders 2008 [10]	n = episodes A-haemolytic Streptococcus(1) Streptococcus group B (agalactiae)(0), S. epidermidis(0), Staphylococcus aureus(0), Stomatococcus rothia mucilaginosa(1), Escherichia coli(1), Pseudomonas aeruginosa(0), Klebsiella pneumoniae(0), non-speciated Gram-negative bacilli(0).	n = episodes A-haemolytic Streptococcus(1) Streptococcus group B (agalactiae)(1), S. epidermidis(3), Staphylococcus aureus(1), Stomatococcus rothia mucilaginosa(0), Escherichia coli(4), Pseudomonas aeruginosa(1), Klebsiella pneumoniae(1), non-speciated Gram-negative bacilli(1).

**Fig. 4:**
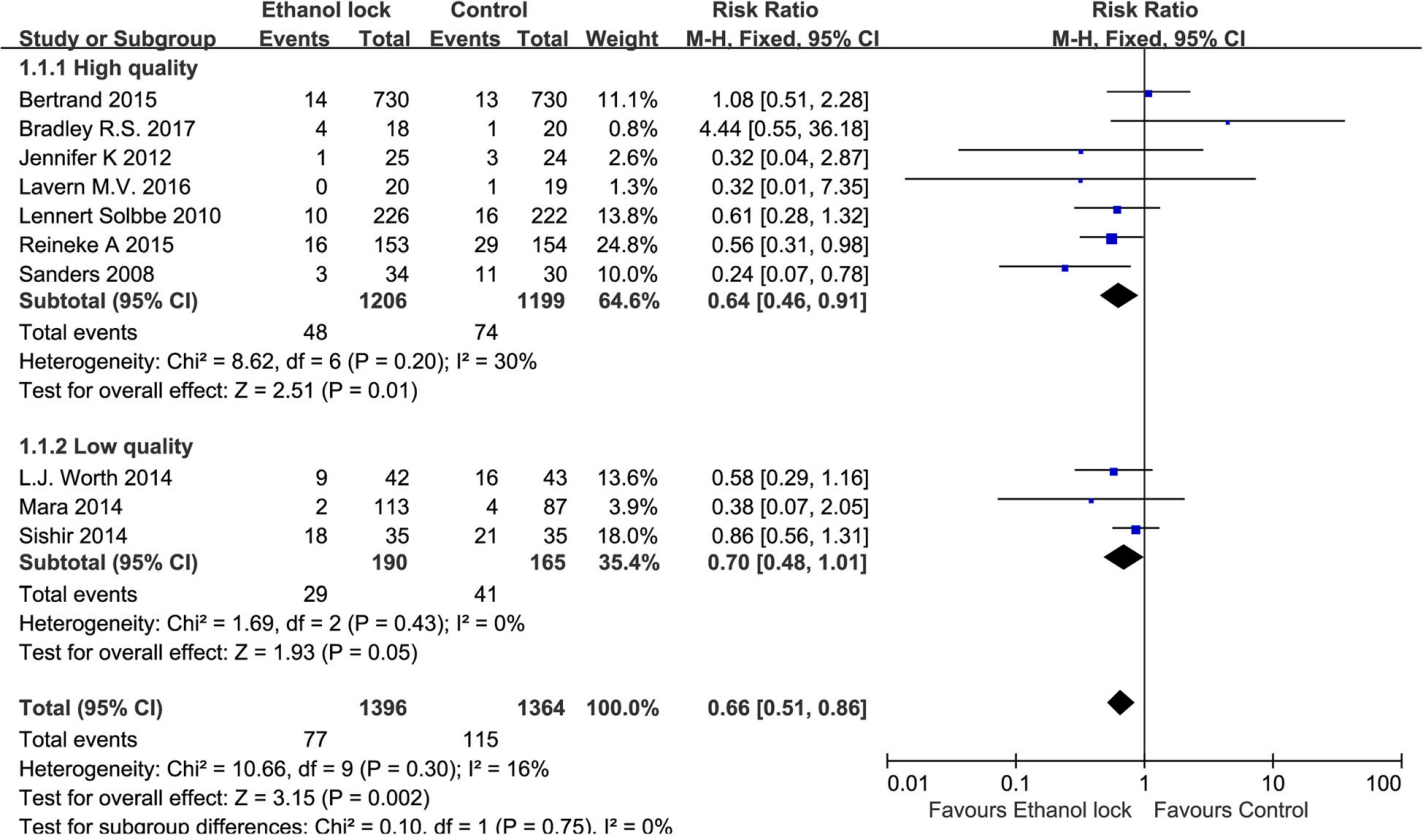
Forest plot for subgroup analysis of the incidence of CRBI according to different study quality (RR, relative risk; CI, confidence interval)

**Fig. 5:**
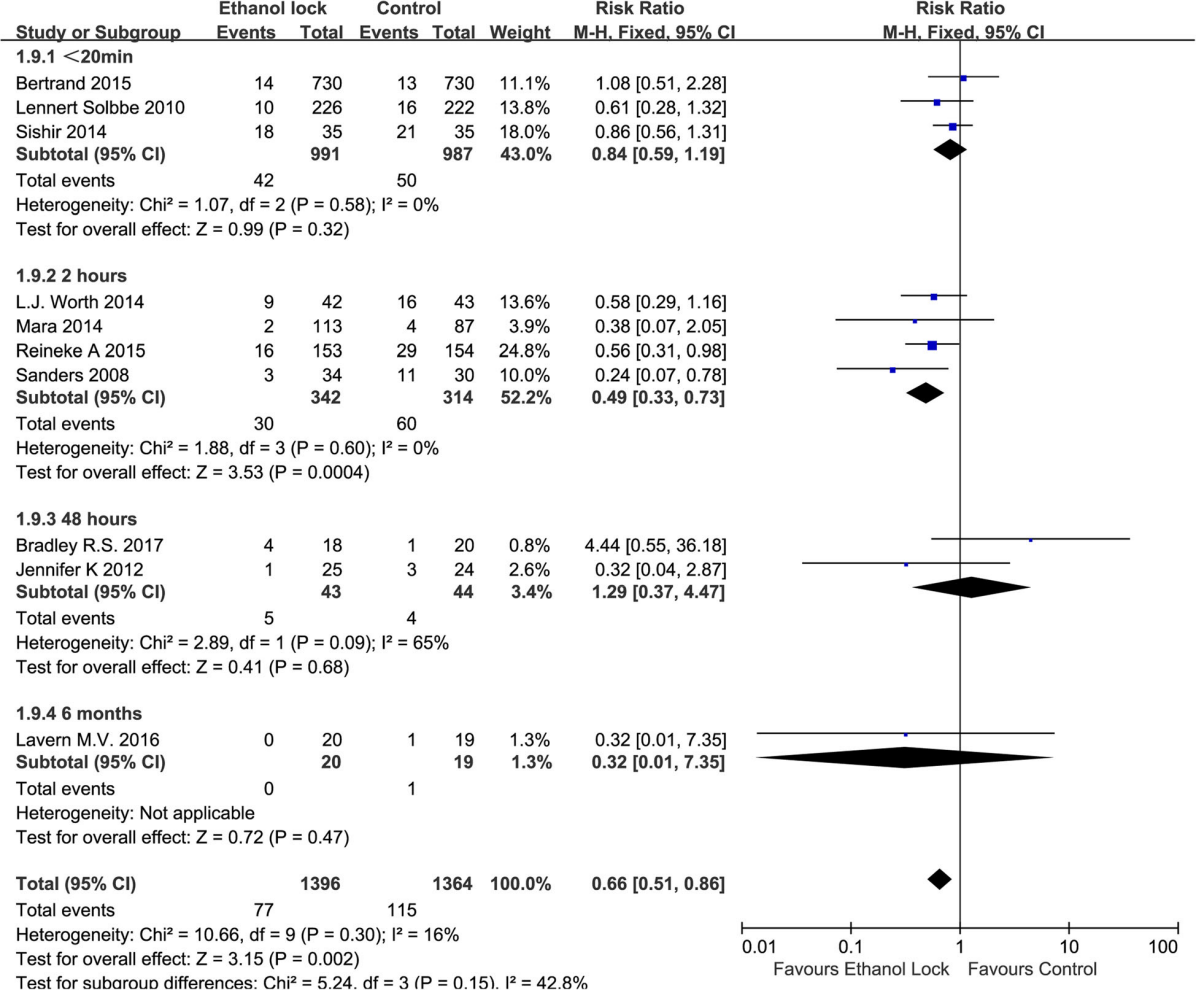
Forest plot for subgroup analysis of the incidence of CRBI according to different ethanol lock duration (RR, relative risk; CI, confidence interval)

**Fig. 6:**
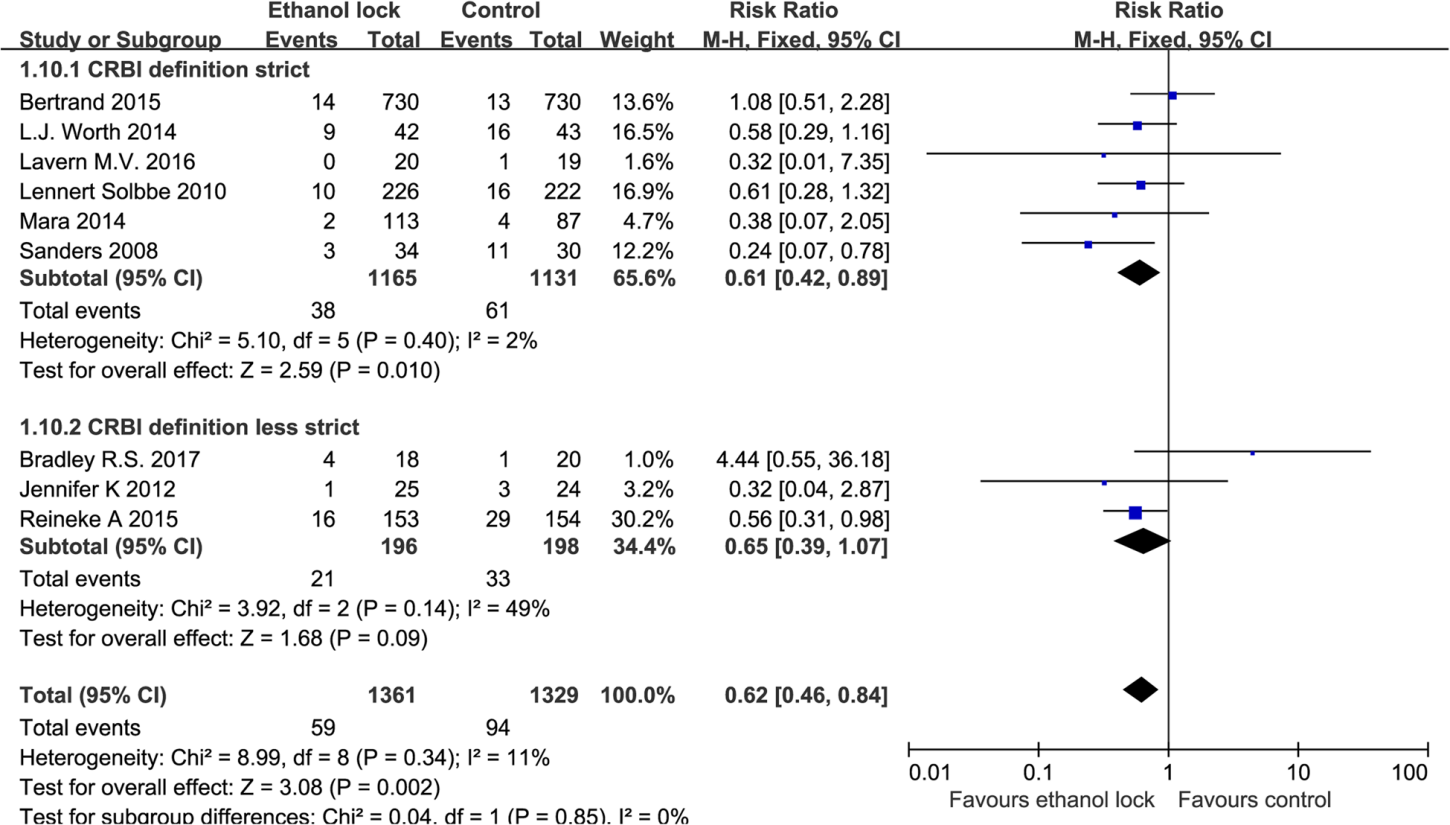
Forest plot for subgroup analysis of the incidence of CRBI according to CRBI definition (RR, relative risk; CI, confidence interval)

Subgroup analysis showed that an ethanol lock can reduce the incidence of CRBI in patients with hematological diseases (RR 0.50, 95% CI 0.31 to 0.80, I^2^ = 0%, Fig. 3). There was no significant difference between ethanol lock and conventional catheter care groups (RR 0.87, 95% CI 0.59 to 1.27) among hemodialysis patients, without significant heterogeneity (I^2^ = 0%, Fig. 3). In addition, an ethanol lock was more effective than traditional controls at preventing CRBI in pediatric oncology patients (RR 0.56, 95% CI 0.31 to 0.98, Fig. 3). Meta-analysis of high-quality studies (random sequence generation, allocation concealment and blinding of participants and personnel in the study can be evaluated as low risk) showed that an ethanol lock significantly reduced CRBI in patients with central venous catheters (RR 0.66, 95% CI 0.47to 0.94),and meta-analysis of low-quality studies also suggested a significant difference in the incidence of CRBI between ethanol lock and control groups (RR 0.66, 95% CI 0.46to 0.95) (Fig. 4). Subgroup analysis indicated that there was a significant difference between 2-h ethanol lock and conventional catheter care groups (RR 0.49 95% CI 0.33 to 0.73), without significant heterogeneity (I^2^ = 0%, Fig. 5). There was no significant difference between less than 20-min ethanol lock and conventional catheter care groups (RR 0.84, 95% CI 0.59 to 1.19), again without significant heterogeneity (I^2^ = 0%, Fig. 5), or48-hour ethanol lock and conventional catheter care groups (RR 1.29, 95% CI 0.37 to 4.47).Meta-analysis of studies with a strict CRBI definition revealed that an ethanol lock can significantly prevent CRBI (RR 0.61, 95% CI 0.42–0.89),though pooled analysis of studies with a less strict CRBI definition suggested no significant change in the incidence of CRBI between ethanol lock and control lock groups (RR 0.65, 95% CI 0.39–1.07) (Fig. 6).

**Fig. 7:**
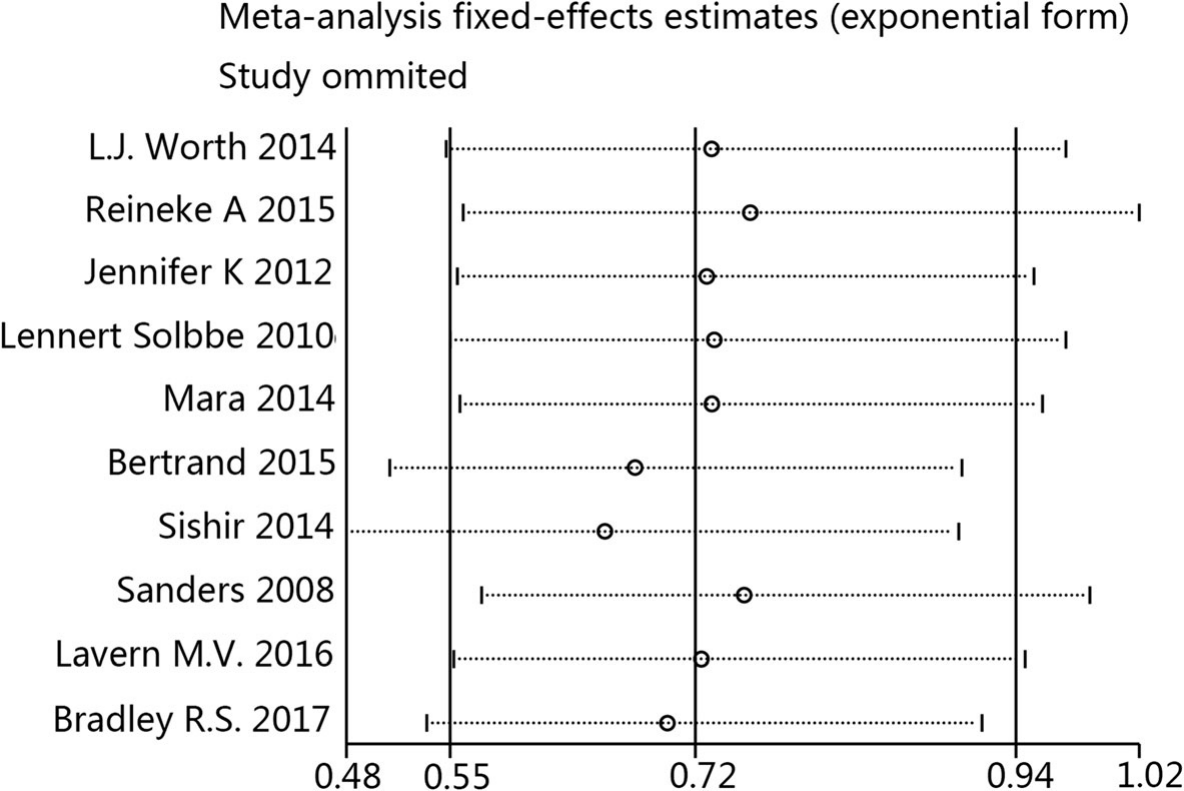
Sensitivity analysis of CRBI results

**Fig. 8:**
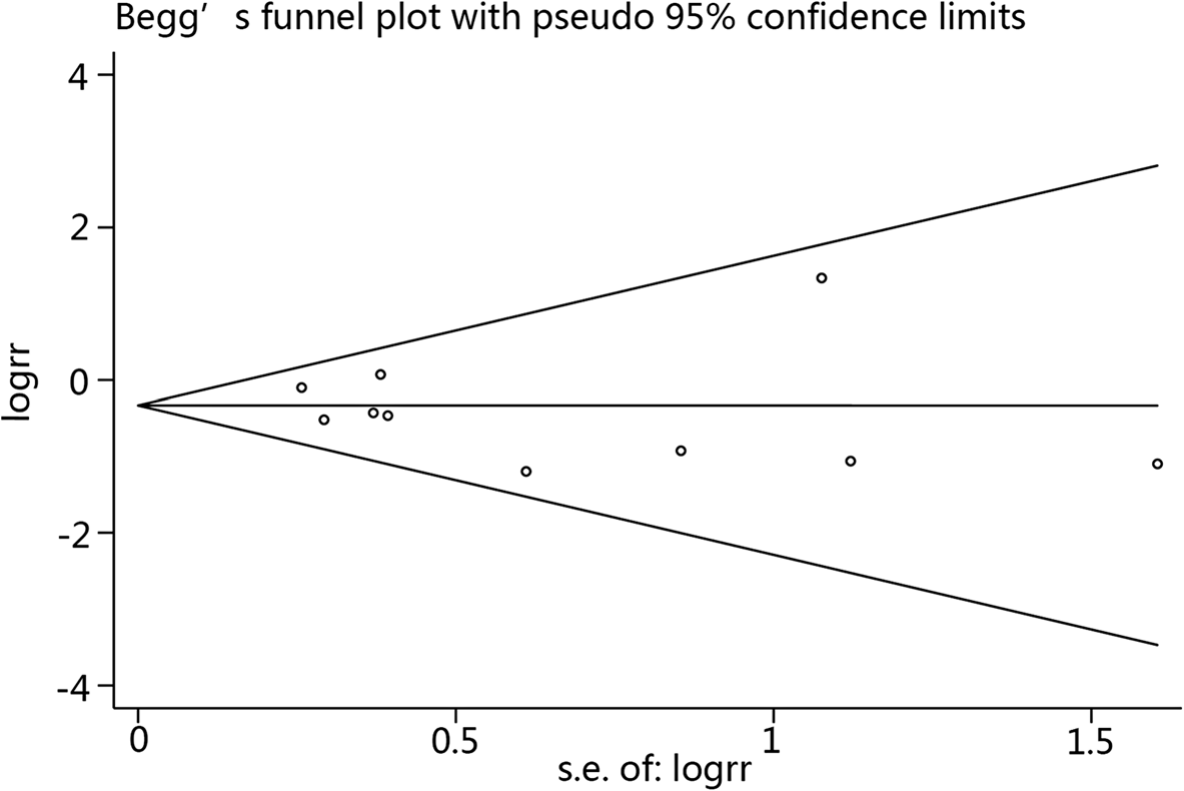
Begg’s funnel plot

Sensitivity analysis results showed that the results were relatively consistent (Fig. 7), and no obvious publication bias was detected, as based on Eggers’ funnel plots (Fig. 8).

### Adverse events

**Fig. 9:**
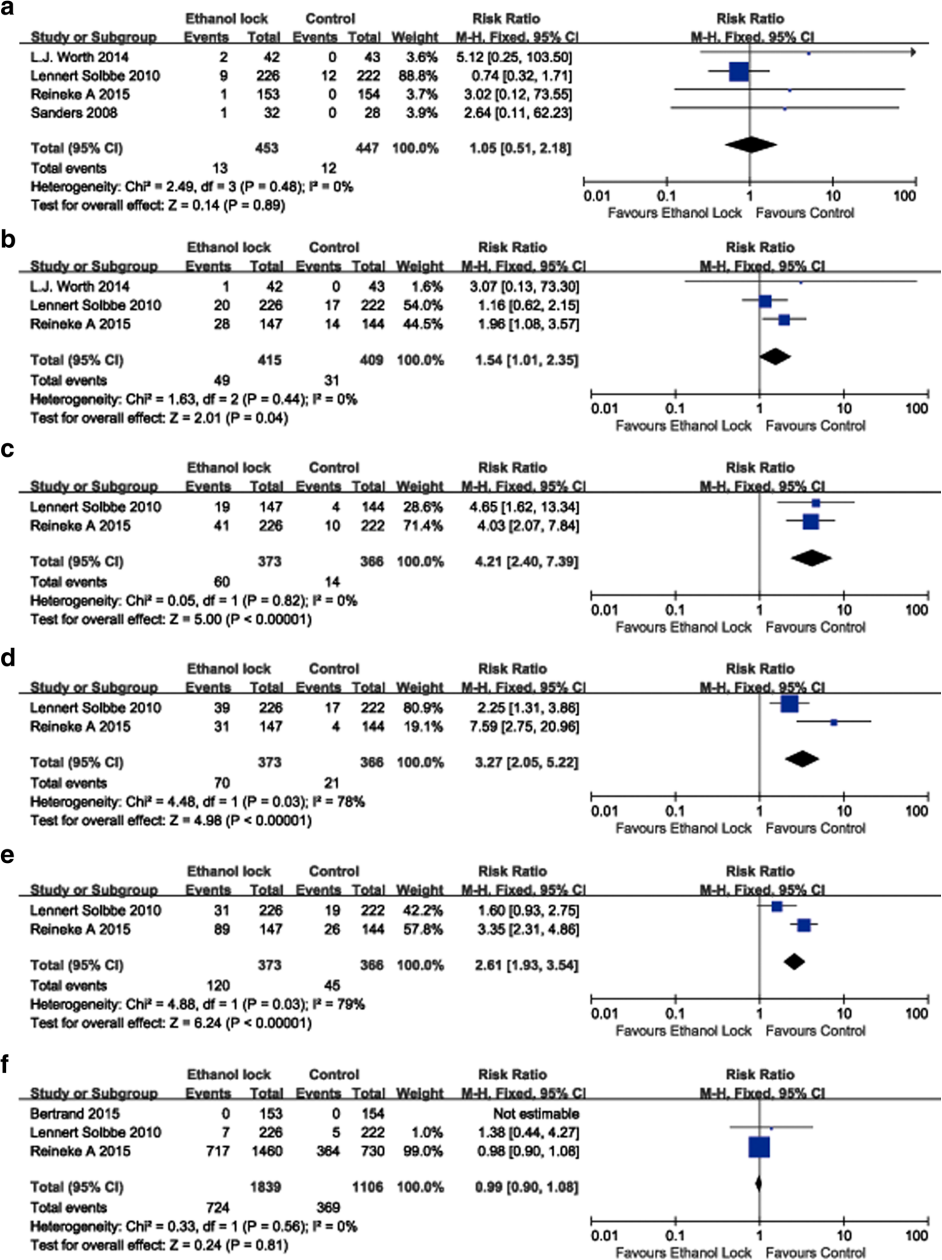
Forest plot for adverse events including thrombus (**a**), nausea (**b**), dizziness (**c**), blushing (**d**), altered taste (**e**) and mortality (**f**)

The results of meta-analysis involving adverse events are depicted in Fig. 7. An ethanol lock did not significantly increase the incidence of a thrombus (RR 1.05, 95% CI 0.51 to 2.18) or mortality (RR 0.99, 95% CI 0.90 to 1.08) but did increase nausea (RR 1.54, 95% CI 1.01 to 2.35), dizziness (RR 4.21, 95% CI 2.40 to 7.39), and blushing (RR 3.27, 95% CI 2.05 to 5.22) and altered taste (RR 2.61, 95% CI 1.93 to 3.54) (Fig. 9).

## Discussion

Our meta-analysis first identified the efficacy of ethanol locks in preventing CRBIs. We found that ethanol locks significantly reduced the incidence of CRBI (RR 0.66, 95% CI 0.51–0.86). Subgroup analysis suggested that an ethanol lock significantly decreased CRBI incidence in patients with hematological diseases (RR 0.50, 95% CI 0.31–0.80), and a meta-analysis that only included high-quality studies showed that an ethanol lock significantly reduced CRBI incidence (RR 0.64, 95% CI 0.46–0.91). A 2-h ethanol lock diminished the frequency of CRBI, but a shorter (less than 20 min) ethanol lock did not decrease infection risk. Additionally, a meta-analysis of studies with strict CRBI definitions showed that an ethanol lock can significantly prevent a CRBI. Although an ethanol lock did not significantly increase thrombus and mortality rates, it did increase certain adverse reactions, such as nausea, dizziness, blushing and altered taste, in patients.

Tunneled CVCs are used for long-term venous access to deliver blood and blood products, chemotherapy and parenteral nutrition. The prevalence of CRBI is high in patients with indwelling CVCs, which also leads to a severe result [19], and internal colonization in long-term tunneled CVCs more frequently contributes to bacteremia [20, 21]. Many methods have been employed to prevent catheter-related sepsis, including the use of cutaneous antisepsis at the time of insertion, catheter tunneling, intraluminal antibiotic locks, antiseptic hubs and anti-microbial coating of catheters [22, 23]. However, these methods may fail to decrease the risk of infection and may instead increase the risk of hypersensitivity and development of anti-microbial resistance. Ethanol-based catheter locks may provide a better alternative because ethanol is a widely used antiseptic with no known acquired resistance [24]. A meta-analysis of observational studies found that ethanol locks are effective alternatives to heparin locks for preventing CRBI in pediatric patients with intestinal failure [25], with the ethanol lock dwell time ranging from more than 2 h per day to 4 h 3 days per week.

To the best of our knowledge, this report describes the first meta-analysis of RCTs to investigate the efficacy of ethanol locks in the prevention of CRBI. Ten RCTs were included in our meta-analysis, and the high quality of the included studies enhances current evidence. Moreover, we performed subgroup analysis based on differences in study quality, duration of the ethanol lock and disease type.

Three studies reported the incidence of CRBI in patients with hematological diseases whose immune system was suppressed, and pooled analysis of these three studies suggested that an ethanol lock significantly reduces CRBI in immunosuppressed patients. Despite no significant difference according to the subgroup analysis in CRBI in hemodialysis patients, immune status or homeostasis may have an effect on the incidence of CRBI with an ethanol lock, which can result in bias among studies. In addition, to exclude bias by differences in study quality, subgroup analysis of relative high-quality or low-quality studies was performed, and the results suggested that an ethanol lock can significantly reduce CRBI risk. However, our definition of high-quality study was different from the Cochran high-quality trial definition; the latter requires all seven domains of the risk of bias assessment tool to be at “low risk of bias”. Consequently, our subgroup analysis results regarding study quality are not very accurate. More high-quality studies that meet the Cochran definition are needed.

Three studies reported that the CVCs were locked with ethanol for 2 min, 15 min, and 20 min. The incidences of CRBI in these studies were determined by pooled analysis, though the short time frame for the ethanol lock did not effectively prevent CRBIs. In addition, the follow-up times were only 48 h, or shorter in the study by Bertrand et al., who used a 2-min ethanol lock. In combination with the low incidence of CRBI in that study, this situation might have contributed to the low efficiency in calculating a difference. In the remaining two studies, ethanol locks exhibited a tendency to prevent CRBIs, though without statistical significance. Interestingly, our findings showed that a 2-h ethanol lock (2-h duration of the lock) significantly decreased the frequency of CRBI. The preferable baseline similarity in the included four studies with a 2-h ethanol lock also enhanced the reliability of our meta-analysis results. Raadet al. found that prolonged exposure to lock solutions containing 25% ethanol in ethylenediaminetetraacetic acid (EDTA) can effectively enhance antibacterial activity in the silicone disk biofilm colonization model [26]. We did not find that a 48-h or 6-month lock can effectively prevent CRBI in our meta-analysis, and this may be due to their smaller size.

The strict definition of CRBI is such that clinical symptoms are not included and only blood culture results are used. Interestingly, meta-analysis of the studies with a strict CRBI definition showed that an ethanol lock can significantly prevent CRBI; however, this was not the result of pooled analysis of studies with a less strict CRBI definition. This finding may be the reason why a less strict CRBI definition reduced the sample weight. Because there was one study that included pediatric patients, the subgroup analysis on age was achieved by sensitivity analysis. Sensitivity analysis showed the results of pooled analysis were relatively stable. Statistical significance was lost when the trial by Reineke et al*.* was removed, which was due to the large sample size (307) of the study, resulting in its larger weight in the pooled result. It is noteworthy that an ethanol lock did not reduce the incidence of mortality, but there was a notable lack of mortality data in most of the trials. No obvious publication bias was detected, enhancing the value of the meta-analysis results. In addition, based on data of the pathogens involved in the infections, we determined that *Staphylococcus* has an important role as a cause of CRBI.

There were also several limitations to our meta-analysis. First, we included only the abstract of studies for which we could not find the full text. Second, although a significant difference was detected in CRBI between ethanol lock and control lock groups according to subgroup analysis, the analyzable number of studies was low, which can result in bias risk. Third, very small differences in catheter type, such as dialysis catheter, and inserting catheters for parenteral nutrition may also lead to bias risk. Fourth, we did not find a significant difference in the incidence of CRBI between ethanol locks and control locks in hemodialysis patients, which was according to meta-analysis results of four studies, and the incidence of CRBI can be influenced by disturbed homeostasis. Sixth, the inclusion of the pediatric population of one study with a large sample might represent a small bias (adult and pediatric populations are different). Finally, patients with an ethanol lock may have certain adverse reactions, such as nausea, dizziness, blushing and altered taste, which might becaused by the ethanol lock solution entering into the bloodstream during catheter use.

## Conclusions

Ethanol locks may play a role in preventing CRBI, though the strength of evidence is limited by the number of studies in the analysis.

## Additional file

Additional file 1: Reasons for the exclusion of 36 studies in the literature screening process. (DOCX 67 kb)

## Abbreviations

CI: Confidence interval
CRBI: Catheter-related bloodstream infection
CVC: Central venous catheters
DCs: Dialysis catheters
MHS: Major Heart Surgery
PRISMA: Preferred reporting items of the systematic review and meta-analyses
RCTs: Randomized controlled trials
RR: Relative risk
