# Supplementary material for: Ethanol locks for the prevention of catheter-related bloodstream infection: a meta-analysis of randomized control trials
Source: BMC Anesthesiol. 2018 Jul 24;18:93. doi: 10.1186/s12871-018-0548-y (PMC6058389; doi:10.1186/s12871-018-0548-y)
Supplement: Supplementary file 1 — Reasons for the exclusion of 36 studies in the literature screening process. (DOCX 67 kb) [file 12871_2018_548_MOESM1_ESM.docx]

**Additional file 1:** **Reasons for the exclusion of 36 studies in the literature screening process.**

**(1)Unmatched intervention(n=1)**

The intervention is not accord with the inclusion standards.

[[1](#_ENREF_1)]

**(2)Published conference papers(n=4)**

[[2-5](#_ENREF_2)]

**(3)Conference papers without the results(n=2)**

[[6](#_ENREF_6), [7](#_ENREF_7)]

**(4)Studied with preliminary results(n=1)**

[[8](#_ENREF_8)]

**(5)Not randomized controlled studies(n=22)**

Retrospective analysis, case control studies, or case series.

[[9-30](#_ENREF_9)]

**(6)Review(n=6)**

[[31-36](#_ENREF_31)]

1. Vercaigne LM, Allan DR, Armstrong SW, Zacharias JM, Miller LM: **An ethanol/sodium citrate locking solution compared to heparin to prevent hemodialysis catheter-related infections: a randomized pilot study**. *Journal Of Vascular Access* 2016, **17**(1):55-62.

2. Broom JK, O'Shea S, Govindarajulu S, Playford EG, Hawley CM, Isbel NM, Campbell SB, Mudge DW, Carpenter S, Johnson BC *et al*: **Rationale and design of the HEALTHY-CATH trial: a randomised controlled trial of Heparin versus EthAnol Lock THerapY for the prevention of Catheter Associated infecTion in Haemodialysis patients**. *BMC nephrology* 2009, **10**:23.

3. Fort AE, Cummings JJ: **A Prospective, Randomized, Blinded, Placebo-Controlled Trial Of Periodic, Brief Ethanol Locks To Prevent Peripherally Inserted Central Catheter (Picc) Infections In Preterm Infants In the Neonatal Intensive Care Unit**. *J Invest Med* 2011, **59**(2):421-422.

4. Pérez MJ, Barrio JM, Rincón C, Hortal J, Martín-Rabadán P, Pernia S, Bouza E: **Ethanol lock therapy in the prevention of catheter-related bloodstream infections after major heart surgery**. *Clinical Microbiology and Infection* 2012, **18**:351.

5. Rijnders BJ, Slobbe L: **Prevention of catheter-related bacteraemia with a daily ethanol-lock in haematology patients with tunnelled catheters. Randomized placebo-controlled trial**. *Clinical Microbiology and Infection* 2010, **16**:S98.

6. Salloum R, McGrath E, Chen X, Becker C, Boldt-Macdonald K, Ang J, Chu R: **Ethanol lock therapy for catheter-related blood stream infections in pediatric patients with a hematologic or oncologic diagnosis**. *Pediatric Blood and Cancer* 2010, **54**(6):827-828.

7. Schoot R, van Ommen CH, Stijnen T, Tissing WJ, Heij H, Lieverst J, Spanjaard L, Caron HN, de Wetering MDV, Aristocaths SS: **Reduction Of Catheter Associated Bloodstream Infections In Paediatric Oncology Patients Using Ethanol Locks; a Randomized Controlled Trial**. *Pediatric blood & cancer* 2014, **61**:S108-S109.

8. Kayton ML, Garmey EG, Ishill NM, Cheung NK, Kushner BH, Kramer K, Modak S, Rossetto C, Hennelly C, Doyle MP *et al*: **Preliminary results of a phase I trial of prophylactic ethanol-lock administration to prevent mediport catheter-related bloodstream infections**. *Journal of pediatric surgery* 2010, **45**(10):1961-1966.

9. Ardura MI, Lewis J, Tansmore JL, Harp PL, Dienhart MC, Balint JP: **Central catheter-associated bloodstream infection reduction with ethanol lock prophylaxis in pediatric intestinal failure: broadening quality improvement initiatives from hospital to home**. *JAMA pediatrics* 2015, **169**(4):324-331.

10. Broom J, Woods M, Allworth A, McCarthy J, Faoagali J, Macdonald S, Pithie A: **Ethanol lock therapy to treat tunnelled central venous catheter-associated blood stream infections: results from a prospective trial**. *Scandinavian journal of infectious diseases* 2008, **40**(5):399-406.

11. Chaudhary M, Bilal M, Du W, Chu R, McGrath E, Rajpurkar M: **Impact of ethanol lock therapy on hospital length of stay and salvage rate in pediatric catheter associated bloodstream infections**. *Pediatric Blood and Cancer* 2013, **60**:S87.

12. Cober MP, Kovacevich DS, Teitelbaum DH: **Ethanol-lock therapy for the prevention of central venous access device infections in pediatric patients with intestinal failure**. *JPEN Journal of parenteral and enteral nutrition* 2011, **35**(1):67-73.

13. Corrigan ML, Pogatschnik C, Konrad D, Kirby DF: **Hospital readmissions for catheter-related bloodstream infection and use of ethanol lock therapy: comparison of patients receiving parenteral nutrition or intravenous fluids in the home vs a skilled nursing facility**. *JPEN Journal of parenteral and enteral nutrition* 2013, **37**(1):81-84.

14. Fort AE, Cummings JJ: **Tolerance of neonatal peripherally inserted central catheters (PICC) TOA 70% ethyl alcohol solution used to prevent infection**. *J Invest Med* 2011, **59**(2):422.

15. Heng AE, Abdelkader MH, Diaconita M, Nony A, Guerraoui A, Caillot N, Rince M, Deteix P, Souweine B: **Impact of short term use of interdialytic 60% ethanol lock solution on tunneled silicone catheter dysfunction**. *Clinical nephrology* 2011, **75**(6):534-541.

16. John BK, Khan MA, Speerhas R, Rhoda K, Hamilton C, Lopez R, Steiger E, Kirby DF: **Ethanol lock therapy in reducing catheter related blood stream infections (CRBSI) in home parenteral nutrition patients**. *Gastroenterology* 2010, **138**(5):S39.

17. Jones BA, Hull MA, Richardson DS, Zurakowski D, Gura K, Fitzgibbons SC, Duro D, Lo CW, Duggan C, Jaksic T: **Efficacy of ethanol locks in reducing central venous catheter infections in pediatric patients with intestinal failure**. *Journal of pediatric surgery* 2010, **45**(6):1287-1293.

18. Kawano T, Kaji T, Onishi S, Yamada K, Yamada W, Nakame K, Mukai M, Ieiri S: **Efficacy of ethanol locks to reduce the incidence of catheter-related bloodstream infections for home parenteral nutrition pediatric patients: comparison of therapeutic treatment with prophylactic treatment**. *Pediatric surgery international* 2016, **32**(9):863-867.

19. Lustig A, Aflalu S: **Novel catheter lock solution in prevention of hemodialysis catheter complications**. *Journal of Clinical Pharmacology* 2011, **51**(9):1341.

20. McGrath EJ, Salloum R, Chen X, Jiang Y, Boldt-MacDonald K, Becker C, Chu R, Ang JY: **Short-dwell ethanol lock therapy in children is associated with increased clearance of central line-associated bloodstream infections**. *Clinical pediatrics* 2011, **50**(10):943-951.

21. Mouw E, Chessman K, Lesher A, Tagge E: **Use of an ethanol lock to prevent catheter-related infections in children with short bowel syndrome**. *Journal of pediatric surgery* 2008, **43**(6):1025-1029.

22. Opilla MT, Kirby DF, Edmond MB: **Use of ethanol lock therapy to reduce the incidence of catheter-related bloodstream infections in home parenteral nutrition patients**. *JPEN Journal of parenteral and enteral nutrition* 2007, **31**(4):302-305.

23. Plourde R, Gothard D, Markowski A, Cockrell E: **Efficacy Of Ethanol Locks for Treatment Of Central Venous Catheter Infections In Pediatric Oncology Patients**. *Pediatric blood & cancer* 2011, **56**(6):940-940.

24. Rajpurkar M, Boldt-Macdonald K, McLenon R, Callaghan MU, Chitlur M, Lusher JM, Becker C: **Ethanol lock therapy for the treatment of catheter-related infections in haemophilia patients**. *Haemophilia : the official journal of the World Federation of Hemophilia* 2009, **15**(6):1267-1271.

25. Takla TA, Zelenitsky SA, Vercaigne LM: **Effectiveness of a 30% ethanol/4% trisodium citrate locking solution in preventing biofilm formation by organisms causing haemodialysis catheter-related infections**. *J Antimicrob Chemoth* 2008, **62**(5):1024-1026.

26. Valentine KM: **Ethanol lock therapy for catheter-associated blood stream infections in a pediatric intensive care unit**. *Pediatr Crit Care Me* 2011, **12**(6):e292-e296.

27. Wales PW, Kosar C, Carricato M, de Silva N, Lang K, Avitzur Y: **Ethanol lock therapy to reduce the incidence of catheter-related bloodstream infections in home parenteral nutrition patients with intestinal failure: preliminary experience**. *Journal of pediatric surgery* 2011, **46**(5):951-956.

28. Raphael B, Gallotto M, McClelland J, Rosa C, Grullon G, Glavin C, Carey AN, Kerr JB, Ozonoff A: **Daily ethanol lock therapy reduces central line-associated bloodstream infections in high-risk home parenteral nutrition patients**. *Journal of pediatric gastroenterology and nutrition* 2016, **63**:S260-S261.

29. Blackwood RA, Issa M, Klein K, Mody R, Willers M, Teitelbaum D: **Ethanol Lock Therapy for the Treatment of Intravenous Catheter Infections That Have Failed Standard Treatment**. *Journal of the Pediatric Infectious Diseases Society* 2017, **6**(1):94-97.

30. Raad I, Chaftari AM, Zakhour R, Jordan M, Al Hamal Z, Jiang Y, Yousif A, Garoge K, Mulanovich V, Viola GM *et al*: **Successful Salvage of Central Venous Catheters in Patients with Catheter-Related or Central Line-Associated Bloodstream Infections by Using a Catheter Lock Solution Consisting of Minocycline, EDTA, and 25% Ethanol**. *Antimicrobial agents and chemotherapy* 2016, **60**(6):3426-3432.

31. Hu Y, Guidry CA, Kane BJ, McGahren ED, Rodgers BM, Sawyer RG, Rasmussen SK: **Comparative effectiveness of catheter salvage strategies for pediatric catheter-related bloodstream infections**. *Journal of pediatric surgery* 2016, **51**(2):296-301.

32. Pitts S, Bergamo D, Cartaya C, Gore B: **Efficacy in the reduction of central line-associated bloodstream infection in a patient with intestinal failure: An ethanol lock pediatric case study**. *JAVA - Journal of the Association for Vascular Access* 2014, **19**(4):217-220.

33. Wolf J, Shenep JL, Clifford V, Curtis N, Flynn PM: **Ethanol lock therapy in pediatric hematology and oncology**. *Pediatric Blood and Cancer* 2013, **60**(1):18-25.

34. Norris LB, Kablaoui F, Brilhart MK, Bookstaver PB: **Systematic review of antimicrobial lock therapy for prevention of central-line-associated bloodstream infections in adult and pediatric cancer patients**. *International journal of antimicrobial agents* 2017, **50**(3):308-317.

35. Rahhal R, Abu-El-Haija MA, Fei L, Ebach D, Orkin S, Kiscaden E, Cole CR: **Systematic Review and Meta-Analysis of the Utilization of Ethanol Locks in Pediatric Patients With Intestinal Failure**. *JPEN Journal of parenteral and enteral nutrition* 2017:148607117722753.

36. **Clinical Application of Prophylactic Ethanol Lock Therapy in Pediatric Patients With Intestinal Failure**. *Gastroenterology nursing : the official journal of the Society of Gastroenterology Nurses and Associates* 2016, **39**(5):E1-E2.
